# Routine baseline clinical data do not reliably predict poor first-year myopia control with low-dose atropine: a machine-learning benchmarking study

**DOI:** 10.3389/fopht.2026.1930814

**Published:** 2026-09-22

**Authors:** Karenna Mehta, Robert A. Clark

**Affiliations:** 1 South Bay Family Eye, Long Beach, CA, United States; 2 Rice University, Houston, TX, United States; 3 Department of Ophthalmology, David Geffen School of Medicine, University of California, Los Angeles, Los Angeles, CA, United States

**Keywords:** low-dose atropine, machine learning, myopia control, pediatric ophthalmology, predictive modeling, risk stratification, treatment response

## Abstract

**Purpose:**

The aim of this study was to determine whether routinely recorded baseline clinical factors, alone or combined with machine-learning predictive modeling, can provide individual risk stratification for myopia control by identifying children who will progress more than 0.25 diopters (D) during their first year of low-dose atropine treatment.

**Methods:**

From a 555-child source cohort treated initially with atropine 0.01% or 0.02%, 468 children had qualifying 9- to 15-month follow-up; 427 children started atropine monotherapy without a baseline optical adjunct. A child was classified as poorly responsive if either treated eye progressed more than 0.25 D of spherical equivalent refraction from baseline to the qualifying visit. This threshold was a practical clinical refraction boundary and was tested in sensitivity analyses using a 0.50-D threshold, annualized progression, and an annualized >0.50 D/year threshold. A prevalence reference, age-plus-refraction logistic regression, elastic-net regression, gradient boosting, and an exploratory multilayer-perceptron neural network were compared by repeated cross-validation.

**Results:**

Of 427 children, 159 (37%) were poorly responsive. Discrimination was poor and machine learning provided no improvement: the area under the curve (AUC) was 0.60 for logistic regression and 0.57 for gradient boosting [difference −0.03; 95% confidence interval (CI) −0.06 to 0.01]. Age alone performed similarly (AUC 0.60). No threshold was clinically useful; a high-sensitivity rule flagged 85% of children to identify 88% of poor responders, with positive predictive value 0.39.

**Conclusions:**

Routinely recorded baseline factors did not provide clinically useful individual-level prediction of the first-year progression category. With this predictor set, observing early on-treatment progression remains the practical way to identify children who may need escalation.

## Introduction

Low-dose atropine is a widely used first-line therapy for progressive childhood myopia (1, 2). Progression on treatment varies, however, and a substantial minority of children are recognized as progressing despite therapy only after months of observation. Because the first-year progression category cannot be known in advance, clinicians typically treat for 6 to 12 months before judging response and deciding whether to escalate. If these poorly responsive children could be identified at treatment initiation, that information could inform consideration of more intensive initial therapy rather than waiting for poor observed control. Prior models have stratified myopia development and high-myopia risk using electronic medical record refraction data and fundus images, and epidemiologic progression data consistently identify younger age as a risk marker, but these studies addressed disease development or natural progression rather than atropine treatment response (3–5). We explored whether routinely recorded baseline clinical factors, alone or combined with machine learning, can identify the children who progress more than 0.25 diopters (D) during the first treatment year and thus might qualify for more intensive treatment at baseline. This analysis does not assume that atropine is the optimal treatment modality for every child; continued progression may reflect natural progression, limited treatment effect, adherence, or a mismatch between the child’s ocular characteristics and the selected treatment. The study tests only the information content of routine baseline variables available in this retrospective dataset, not richer longitudinal, biometric, optical, familial, behavioral, environmental, or genetic prediction frameworks.

## Methods

### Study design and cohort

This retrospective study was approved by the Providence Institutional Review Board with a waiver of informed consent, adhered to the tenets of the Declaration of Helsinki, and complied with the Health Insurance Portability and Accountability Act of 1996. We analyzed a single-practice cohort that was the source cohort of a companion analysis (6). The source cohort was drawn from 787 charts of children prescribed low-dose atropine for progressive myopia. Exclusions in the source cohort were no follow-up, insufficient follow-up, never starting or persistently inconsistent use that precluded meaningful interpretation, orthokeratology with missing spherical equivalent refraction (SER) data, prior long-term atropine 1%, congenital glaucoma, Down syndrome, and Marfan/Ehlers-Danlos syndromes. The resulting source cohort included 555 children treated with atropine 0.01% (*n* = 519) or 0.02% (*n* = 36) and followed for at least 12 months. For the present first-year prediction analysis, 468 children had a qualifying 9- to 15-month visit for all treated eyes and complete routinely recorded baseline predictors; 427 remained in the primary cohort after excluding 41 children who received a baseline optical adjunct. In this analysis, child refers to one pediatric patient, combining both treated eyes into one child-level record when both eyes were treated. The primary cohort had baseline age 0.4–18.7 years, baseline SER in the more myopic eye from −18.12 to +0.38 D, and baseline cylinder from 0 to 6.00 D in absolute magnitude; 71 children (16.6%) had cylinder ≥1.5 D at baseline.

### Outcome

A child was poorly responsive if either treated eye showed first-year SER progression and baseline SER minus SER at the qualifying visit exceeded 0.25 D. The 0.25-D threshold corresponds to one standard clinical refraction increment and should be interpreted as a practical action boundary rather than a biologically validated breakpoint.

### Predictors

Candidate predictors, all recorded at the initial visit, were baseline age, SER in the more myopic eye, sex, high astigmatism (refractive cylinder ≥1.5 D), anisometropia, retinopathy of prematurity (ROP), and amblyopia; binary predictors were positive if present in either eye. Because a baseline optical adjunct is a simultaneous treatment decision and may reflect clinician assessment of treatment-modality needs, the primary cohort comprised children starting atropine monotherapy; a full-regimen sensitivity analysis added the adjunct as a covariate.

### Models

Five models were compared: a prevalence reference, age-plus-SER logistic regression, elastic-net regression, gradient boosting using XGBoost (eXtreme Gradient Boosting) (7) with shallow trees, and a multilayer-perceptron neural network. The age-plus-SER model was intentionally sparse because prior progression models identify age as a dominant routinely available predictor (5), but the elastic-net, gradient-boosting, and neural-network models also incorporated all seven deployable baseline variables available in the dataset.

### Neural-network benchmark

The neural network was added after the locked primary analysis as an exploratory capacity benchmark, not as a primary claim of deep-learning added value. Given the modest sample size (427 children, 159 events) and seven routinely recorded predictors, it was restricted to a single hidden layer with weight decay and used the same in-fold standardization and out-of-fold evaluation as the other models.

### Validation and performance metrics

Models were evaluated by 10 repeats of fivefold cross-validation, reporting out-of-fold area under the curve (AUC) with bootstrap 95% confidence intervals (CIs), Brier scores, and calibration. We report the simple model’s sensitivity, specificity, and positive predictive value (PPV) using thresholds chosen within training folds (a balanced threshold and a high-sensitivity threshold targeting training sensitivity ≥0.90) applied to held-out children.

### Sensitivity analyses and interpretability

Prespecified sensitivity analyses used a 0.50-D threshold, an annualized (D/year) threshold, continuous progression, exclusion of early escalators, and the full-regimen cohort. *Post-hoc* sensitivity checks examined age alone, an annualized >0.50 D/year threshold, restriction to children starting atropine 0.01% only, exclusion of children with ROP, restriction to ages 6–18 years, and descriptive performance within baseline age strata (<8, 8–12, and >12 years). For descriptive interpretability, SHAP (SHapley Additive exPlanations) values (8) were calculated from the gradient-boosting model fitted to the full cohort; these feature-importance estimates describe how that model used the available predictors and were not treated as independently validated predictive effects. Reporting follows TRIPOD+AI (9); the protocol was locked after data-structure review and is exploratory.

## Results

Of 427 children, 402 (94%) started atropine 0.01% and 25 (6%) started 0.02%. A total of 159 (37%) met the poorly responsive classification, including 86 (20%) with this classification in both eyes. Twenty-four children (5.6%) had a history of ROP; these children were retained because the study was intended to reflect the full referral-practice population and because ROP status was available as a baseline covariate. Children meeting the poorly responsive classification were younger than responsive children (mean 9.1 vs. 10.1 years) and more often had amblyopia (36.5% vs. 25.7%); baseline SER and the remaining characteristics were similar (**Table 1**). Discrimination was poor, with no improvement from machine learning (**Table 2**; **Figure 1**): the AUC was 0.60 (95% CI 0.54–0.65) for age-plus-SER logistic regression, 0.56 for elastic-net regression, and 0.57 (0.52–0.63) for gradient boosting, and 0.56 for a multilayer-perceptron neural network; no model improved on age-plus-SER (largest difference −0.03, 95% CI −0.06 to 0.01; **Table 1**). Age alone performed similarly to age plus SER (AUC 0.60; 95% CI 0.55–0.66). In the age-and-SER model, each additional year of age lowered the odds of meeting the poorly responsive classification (odds ratio 0.87; 95% CI 0.81–0.94; *p* < 0.001), whereas baseline SER was not associated with this classification (odds ratio 0.96; *p* = 0.33). Descriptive full-cohort SHAP analysis of the gradient-boosting model ranked age and baseline SER as the largest contributors, with much smaller contributions from the remaining variables.

**Table 1:** Baseline characteristics by first-year myopia control.

Characteristic	Good control	Poor control	Overall
Children, *n*	268	159	427
Baseline age, y, mean (SD)	10.1 (2.7)	9.1 (2.8)	9.8 (2.8)
Baseline SER, D, mean (SD)	−2.5 (2.5)	−2.7 (2.2)	−2.5 (2.4)
Female, *n* (%)	140 (52.2)	83 (52.2)	223 (52.2)
ROP, *n* (%)	16 (6.0)	8 (5.0)	24 (5.6)
Amblyopia, *n* (%)	69 (25.7)	58 (36.5)	127 (29.7)
High astigmatism, *n* (%)	42 (15.7)	29 (18.2)	71 (16.6)
Anisometropia, *n* (%)	39 (14.6)	22 (13.8)	61 (14.3)
Complete baseline predictors, *n* (%)	268 (100)	159 (100)	427 (100)

A child had poor control if either treated eye had spherical equivalent refraction (SER) worsening exceeding 0.25 D from baseline to the qualifying visit, and good control otherwise. Values are mean (standard deviation) for continuous variables and number (percentage) for categorical variables. D, diopters; ROP, retinopathy of prematurity; SD, standard deviation; y, years.

**Table 2:** Out-of-fold model performance for predicting child-level poor first-year myopia control.

Model	AUC (95% CI)	Brier	Calibration intercept	Calibration slope
M0 prevalence reference	—	0.235	—	—
M1 age + SER logistic	0.60 (0.54–0.65)	0.229	0.00	0.77
M2 elastic-net	0.56 (0.51–0.61)	0.232	0.00	1.30
M3 gradient boosting	0.57 (0.52–0.63)	0.233	0.00	0.51
M_NN multilayer perceptron	0.56 (0.51–0.61)	0.241	0.00	0.29
M3 − M1 difference	−0.03 (−0.06 to 0.01)	—	—	—

Ten repeats of fivefold cross-validation were performed. Brier score, calibration intercept, and calibration slope are based on one cross-validated prediction per child, obtained by averaging that child’s 10 held-out probabilities across repeated cross-validation; calibration intercepts were |·| < 0.005 (predictions centered near the outcome prevalence) and are shown rounded to two decimals. AUC, area under the curve; CI, confidence interval; M_NN, multilayer perceptron; SER, spherical equivalent refraction; —, not applicable.

**Figure 1:**
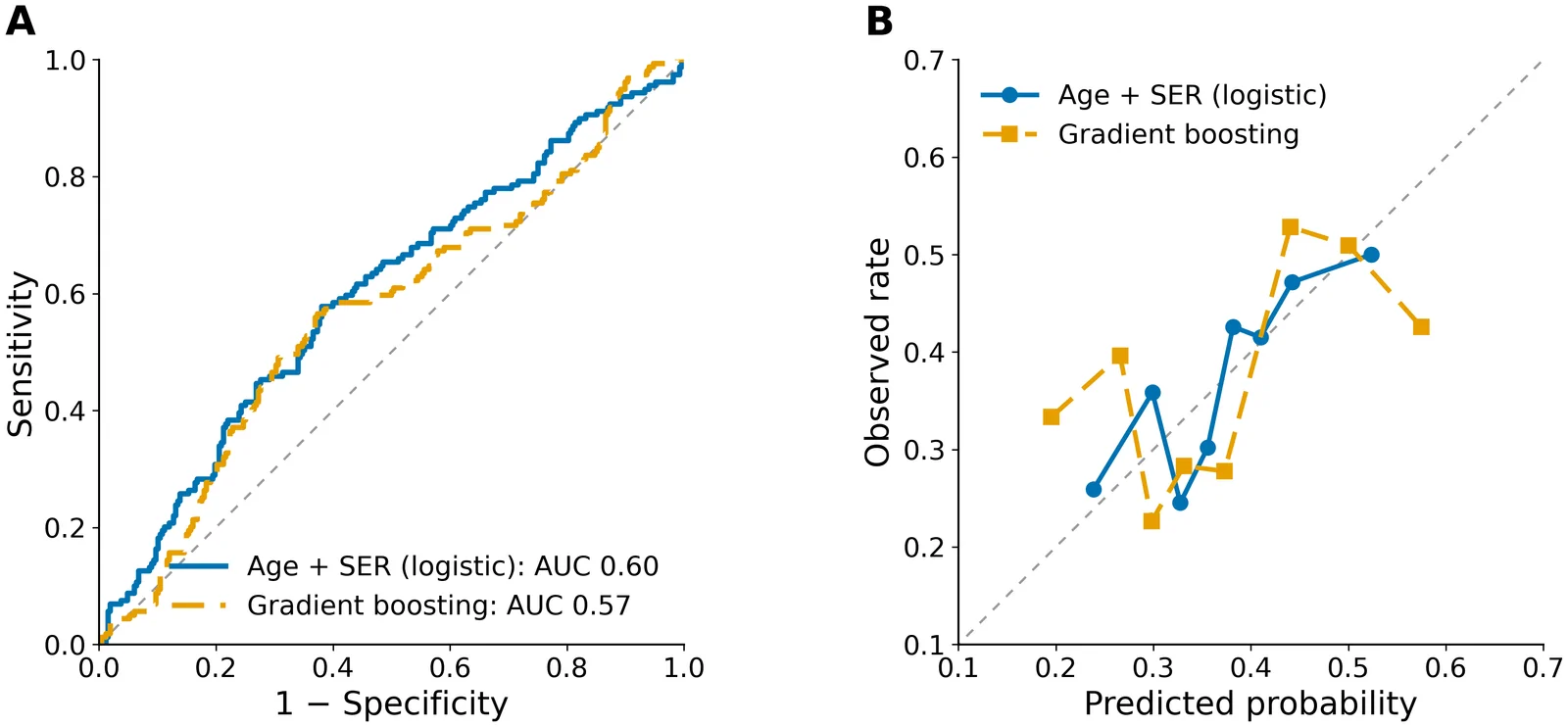
Discrimination and calibration for predicting poor first-year myopia control. **(A)** Receiver-operating-characteristic curves for the age-plus-SER logistic model (solid) and the gradient-boosting model (dashed), with out-of-fold areas under the curve; the diagonal denotes chance. **(B)** Reliability (calibration) plot of observed versus predicted probability for the same two models; the dashed diagonal indicates perfect calibration, and predictions cluster within a narrow band near the outcome prevalence. In **(B)**, points represent grouped quantiles (octiles) of the per-child averaged held-out predicted probabilities. AUC, area under the curve; SER, spherical equivalent refraction.

No operating threshold identified poorly responsive children well enough to guide treatment at initiation (**Table 3**). At a balanced threshold, flagging 45% of children identified 57% of those actually poorly responsive, and most flagged children responded well (PPV 0.47). At a high-sensitivity threshold, identifying 88% of poorly responsive children required flagging 85% of all children, with PPV 0.39 against a 37% failure rate. Results were unchanged at a 0.50-D threshold (logistic AUC 0.56), under an annualized definition (0.58), using an annualized >0.50 D/year threshold (0.57), with progression modeled continuously (cross-validated *R*^2^ ~ 0), after excluding early escalators (0.58), and in the full-regimen cohort (0.60). *Post-hoc* sensitivity analyses were similar when restricted to children starting atropine 0.01% only (*n* = 402; AUC 0.61), after excluding children with ROP (*n* = 403; AUC 0.59), and when restricted to children aged 6–18 years (*n* = 396; AUC 0.58). Descriptive age-stratum analyses did not suggest hidden clinical utility, with age-plus-SER AUCs of 0.52 for children younger than 8 years, 0.54 for those 8–12 years, and 0.54 for those older than 12 years.

**Table 3:** Operating characteristics of age and baseline spherical equivalent refraction.

Operating point	Sensitivity	Specificity	PPV	% flagged
Balanced (Youden)	0.57	0.62	0.47	45
High-sensitivity (≥0.90)	0.88	0.17	0.39	85

Predictive value of the simple (age and baseline spherical equivalent refraction) model for flagging children with poor first-year control, using decision thresholds selected within training folds (prevalence 37.2%). Balanced (Youden), threshold maximizing the Youden index (sensitivity + specificity − 1); high-sensitivity, threshold targeting a training sensitivity ≥ 0.90. PPV = positive predictive value.

## Discussion

Routinely recorded baseline clinical factors, alone or combined with machine learning, could not identify children at treatment initiation who later progressed more than 0.25 D during the first year of low-dose atropine. Gradient boosting did not exceed a two-variable age-and-refraction model, and the single weak association, younger age with greater first-year progression, is compatible with the expected slowing of myopic progression with age (5) and cannot, without an untreated comparator, be separated from differential treatment response. The model also cannot determine whether continued progression reflects insufficient atropine effect, poor adherence, natural progression, or a better match to another modality such as peripheral-defocus spectacles or contact lenses. Consistent with this, the more flexible models were also more poorly calibrated (calibration slope 0.51 for gradient boosting and 0.29 for the neural network, versus 0.77 for the two-variable model), indicating that added model capacity produced overfitting rather than recovered signal.

These baseline factors therefore do not support assigning children to intensified initial therapy or to an alternative treatment modality at baseline. For this predictor set, measurement of early on-treatment progression remains the practical risk-stratification step for identifying children who may need escalation or modality change, complementing the companion analysis (6) in which the observed 12-month response was itself an actionable escalation signal. This conclusion should not be read as implying that early follow-up is the only possible risk-stratification strategy; future models incorporating longitudinal, biometric, optical, familial, behavioral, environmental, or genetic data may perform differently.

One response to this null result might be to start every child on maximum therapy or combined therapy; the evidence argues against unselective intensification. In the Childhood Atropine for Myopia Progression (CHAMP) trial (10), higher-concentration atropine did not improve refractive outcomes (0.01% outperformed 0.02%), and escalating already responsive children in this cohort did not slow progression (6), whereas universal intensification adds dose-related toxicity and cost for the majority well controlled on the lower dose. The inability to identify poorly responsive children in advance from routine baseline variables therefore supports observing the initial response to treatment and escalating or changing modality when necessary, not starting all children with maximal or combined treatment.

Strengths include a clinically aligned, child-level outcome, subject-level validation, threshold selection within training folds, and inclusion of all routinely recorded baseline variables available in the dataset. Limitations include the single-center retrospective design, partly non-cycloplegic refractions, the absence of an untreated control group, and internal validation only. The 0.25-D primary threshold is close to the resolution of routine clinical refraction, so children near the boundary may have been misclassified; the similar findings using a 0.50-D threshold, annualized thresholds including >0.50 D/year, and continuous progression reduce but do not eliminate this concern. Requiring a qualifying 9- to 15-month visit may also have selected children with better follow-up adherence or other clinical characteristics, and the resulting cohort should not be assumed to represent all children starting atropine. Children with ROP were retained to preserve the referral-practice cohort, but their ocular anatomy and myopia mechanisms may differ from uncomplicated juvenile myopia; ROP status was included as a candidate predictor, and the small ROP subgroup precludes a separate validated model. Initial atropine concentration was not randomized, and the 0.02% subgroup was small; therefore, concentration-specific treatment effects could not be estimated reliably. Axial length, corneal curvature, peripheral refraction, accommodative lag, pretreatment progression rate, parental myopia, time outdoors, near work, adherence before the endpoint, and genetic or biomarker data were unavailable. Refractive outcomes alone may miss early axial elongation or treatment-modality mismatch. The negative result is therefore bounded: these routinely recorded baseline variables contained insufficient predictive information, but the result should not be generalized to models using richer longitudinal, biometric, optical, familial, behavioral, environmental, or biological data. The null does not appear to reflect limited power or thresholding: 159 events across seven candidate predictors provided approximately 23 events per variable, and continuous progression was itself unpredictable (cross-validated *R*^2^ ~ 0).

## Data Availability

The data analyzed in this study is subject to the following licenses/restrictions: The datasets are not readily available because of institutional restrictions; requests to access should be directed to the corresponding author. Requests to access these datasets should be directed to drraclark@yahoo.com.
